# Enhanced anti-glioma activity of annonaceous acetogenins based on a novel liposomal co-delivery system with ginsenoside Rh2

**DOI:** 10.1080/10717544.2024.2324716

**Published:** 2024-03-31

**Authors:** Hui Ao, Huizhu Song, Jing Li, Xiangtao Wang

**Affiliations:** a Department of Pharmacy, The Affiliated Wuxi People’s Hospital of Nanjing Medical University, Wuxi People’s Hospital, Wuxi Medical Center, Nanjing Medical University, Wuxi, PR China; b Institute of Medicinal Plant Development, Chinese Academy of Medical Sciences & Peking Union Medical College, Beijing, PR China

**Keywords:** Annonaceous acetogenins, Rh2, co-loaded liposomes, anti-glioma, systemic toxicity

## Abstract

Annonaceous acetogenins (ACGs) have potent anti-tumor activity, and the problems of their low solubility, hemolysis, and *in vivo* delivery have been solved by encapsulation into nanoparticles. However, the high toxicity still limits their application in clinic. In this paper, the co-delivery strategy was tried to enhance the *in vivo* anti-tumor efficacy and reduce the toxic effects of ACGs. Ginsenoside Rh2, a naturally derived biologically active compound, which was reported to have synergistic effect with paclitaxel, was selected to co-deliver with ACGs. And due to its similarity with cholesterol in chemical structure, the co-loading liposomes, (ACGs + Rh2)-Lipo, were successfully constructed using Rh2 instead of cholesterol as the membrane material. The obtained (ACGs + Rh2)-Lipo and ACGs-Lipo had similar mean particle size (about 80 nm), similar encapsulation efficiency (EE, about 97%) and good stability. The MTS assay indicated that (ACGs + Rh2)-Lipo had stronger toxicity *in vitro*. In the *in vivo* study, in contrast to ACGs-Lipo, (ACGs + Rh2)-Lipo demonstrated an improved tumor targetability (3.3-fold in relative tumor targeting index) and significantly enhanced the antitumor efficacy (tumor inhibition rate, 72.9 ± 5.4% vs. 60.5 ± 5.4%, *p* < .05). The body weight change, liver index, and spleen index of tumor-bearing mice showed that Rh2 can attenuate the side effects of ACGs themselves. In conclusion, (ACGs + Rh2)-Lipo not only alleviated the toxicity of ACGs to the organism, but also enhanced their anti-tumor activity, which is expected to break through their bottleneck.

## 1. Introduction

Annonaceous acetogenins (ACGs) are a class of long-chain fatty acid lactones derived from the seeds of *Annona squamosa*, and have potent anti-tumor activity and ability to reverse multidrug resistance, which received widespread attention (Aguilar-Hernández et al., 2020; Manoharan et al., 2023). The powerful anti-tumor activity of ACGs stems from their unique anti-tumor mechanism. By acting on mitochondria in cells, ACGS inhibits NADH-ubiquinone oxidoreductase in the mitochondrial respiratory chain, making electron transfer blocked and unable to produce the energy-supplying substance ATP, thus cutting off the energy source of cells (Ohta et al., 2022; Schiller & Zickermann, 2022). This particular mechanism of action has a great impact on the rapidly proliferating tumor cells. Our research team prepared dozens of ACGs-loaded regular and long-circulating nanoparticles using amphiphilic polymer materials, which demonstrated improved solubility and enhanced anti-tumor activity, and solved the problem of low solubility, hemolysis and difficulty of *in vivo* delivery for ACGs (Hong, Li, Li, et al., 2016; Hong, Li, Xiao, et al., 2016; Hong et al., 2017; Li et al., 2018). However, the main problems currently limiting the widespread use of ACGs are their high toxicity. For some tumor types, low doses of chemotherapy drugs could not effectively inhibit tumor growth, but at high doses, they could easily lead to death of tumor-bearing mice. Screening for tumor cell lines that are extremely sensitive to ACGs or by combination administration can help reduce the dose administered and mitigate side effects, which is expected to address the barriers that limit the clinical application of ACGs.

The occurrence and progression of cancer often involve multiple signaling pathways and multiple targets. In the treatment process, monotherapy can achieve good curative effect in the early stage, but in the later stage, the tumor will activate the resistance mechanism and lead to the occurrence of drug resistance (Han et al., 2023). Besides, the chemotherapy drugs are mostly cytotoxic drugs, which will cause more serious side effects (Deepak et al., 2023; Peña-Corona et al., 2023). Therefore, combined administration has attracted more and more attention in anti-tumor research, which can not only reduce the occurrence of drug resistance, improve therapeutic efficacy, but also reduce the incidence of side effects by acting on multiple targets (Li et al., 2023; Nguyen et al., 2023; Silva et al., 2023). Our research group has attempted to combine liver-protective drugs (such as glycyrrhizin, glycyrrhetinic acid, oleanolic acid, diammonium glycyrrhizinate, silymarin, and puerarin) to counteract the toxic effect of ACGs, but no combination has been found that can reduce the toxicity while maintaining the efficacy, and the combination almost invariably significantly reduced the anti-tumor effect of ACGs *in vivo* and *in vitro*. Searching for chemotherapeutic drugs or non-cytotoxic drugs that have synergistic effect with ACGs can bring new ideas for the use of ACGs in oncology treatment.

Ginsenosides derived from the Chinese traditional medicine ginseng, have biological activities such as anti-tumor, immune regulation, treatment of nervous system diseases and cardiovascular diseases (Huang et al., 2021; Lu et al., 2023; Peng et al., 2023; Wang et al., 2023), among which anti-tumor activity has been confirmed by a large number of studies (Jin et al., 2018; Lyu et al., 2019; Chen et al., 2021), and ginsenoside Rg3 has been clinically used in the synergistic treatment of liver cancer and lung cancer. There are also many studies on ginsenosides combined chemotherapy drugs. Rg1 can effectively reduce the cardiotoxicity of doxorubicin (DOX) and enhance its inhibitory effect on 4T1 cells by self-assembly loading of DOX (Li et al., 2021). Rd can reverse the resistance of non-small cell lung cancer A549 cells to cisplatin (Chian et al., 2019). In addition, ginsenosides are triterpenoid saponins with skeleton structure similar to cholesterol, which can be used to replace cholesterol as membrane material to prepare liposomes loaded with chemotherapy drugs (Hong et al., 2020; Zhu et al., 2021; Xia et al., 2022). The cholesterol-free liposomes co-loaded with ginsenoside Rh2 and paclitaxel (PTX) prepared by Hong et al. (2020) displayed four advantages compared to conventional liposomes: (1) the stability of the liposomes was ensured while avoiding cholesterol-induced disadvantages such as hyperlipidemia and pulmonary hypertension; (2) the glucose contained in the Rh2 structure could reduce the phagocytosis of mononuclear phagocyte system (MPS) and prolong the blood circulation time; (3) the glucose exposed on the surface of liposomes can be recognized and bind with glucose transporters highly expressed on the surface of tumor cells, thus enhancing the uptake of drug-loaded liposomes by tumor cells; (4) Rh2 can reshape the tumor microenvironment and reverse the immunosuppressive effects.

**Scheme 1.**
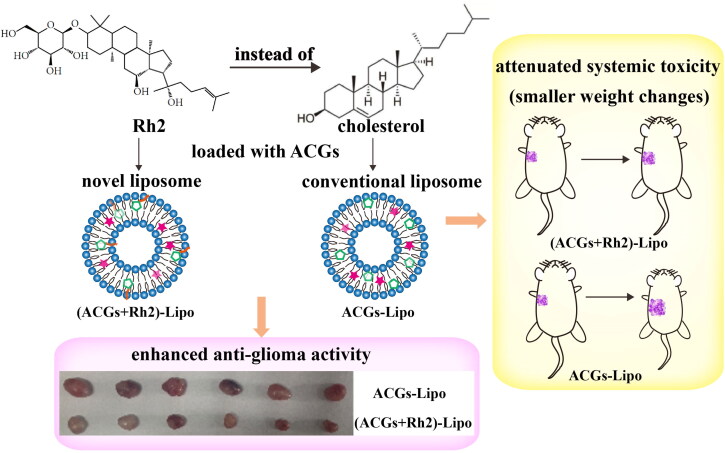
Schematic illustration of a novel liposomal co-delivery system with ginsenoside Rh2 for enhancing anti-glioma activity and attenuate systemic toxicity (all details on the picture were created by the author Hui Ao).

In the study, U87 MG, a tumor cell line that is extremely sensitive to ACGs, was first screened with a half inhibitory concentration (IC_50_) value of ng/mL level, which is expected to maintain strong anti-tumor activity at lower doses. Subsequently, Rh2 instead of cholesterol was loaded with ACGs to construct co-loaded liposomes, and anti-tumor studies on U87 MG cells were conducted *in vivo* and *in vitro*. The results demonstrated that ACGs and Rh2 have good synergistic anti-tumor activity, and co-loaded liposomes enhanced anti-glioma activity and tumor targeting ability, and reduce the toxic effects of ACGs (Scheme 1).

## 2. Materials and methods

### 2.1. Materials

ACGs were obtained from Professor Jianyong Si’s laboratory (Institute of Medicinal Plant Development (IMPLAD), Beijing, China). Rh2 was purchased from Nanjing Spring & Autumn Biotech Co., Ltd. (Nanjing, China). Soybean phospholipids (SPCs) were provided by Shenyang Tianfeng Biological Pharmaceutical Co., Ltd. (Shenyang, China). Cholesterol was purchased from Shanghai Yuanye Bio-Technology Co., Ltd. (Shanghai, China). 1,1-Dioctadecyl-3,3,3,3-tetramethylindotricarbocyanine iodide (DiR) was supplied by AAT Bioquest Inc. (Sunnyvale, CA). Temozolomide (TMZ) was purchased from Dalian Meilun Biological Technology Co., Ltd. (Dalian, China). All the other reagents were of analytical grade or higher. The water used was deionized in the research.

### 2.2. Cell lines and animals

U87 MG cell lines (RRID: CVCL_0022) were supplied by China infrastructure of cell line resource and cultured in MEM medium supplemented with 10% fetal calf serum (FBS) and 100 U/mL penicillin and streptomycin (Gibco, St. Louis, MO) with 5% CO_2_ (Thermo 311, Waltham, MA) at 37 °C.

Thirty-five female BALB/c nude mice (20 ± 2 g, 6–8 weeks old) were purchased from Vital River Laboratory Animal Technology Co., Ltd. (Beijing, China). All mice were cared according to the feeding standards for experimental animals. All animal experiments were performed with reference to the protocol for animal experiments as defined by the Institute of Medicinal Plant Development (IMPLAD), Beijing, China. Ethical approval for this study was granted by the ethics committee of IMPLAD.

### 2.3. Preparation of ACGs-Lipo

ACGs-Lipo was prepared by stirring-dropping method and the optimal formulation was the mass ratio of 30:5:2 (SPC:cholesterol:ACGs) with the concentration of SPC being 10 mg/mL. In detail, the mixture of SPC, cholesterol and ACGs was co-dissolved in 2 mL anhydrous ethanol and injected into deionized water under stirring condition (300 rpm/min, Shanghai Sile Instrument Co., Ltd., Shanghai, PR China) at 25 °C. After infusion, the solution was continued to stir for 2.5 h, and ACGs-Lipo was obtained after the organic solvent was removed by evaporation at 45 °C.

### 2.4. Preparation of (ACGs + Rh2)-Lipo

The method of thin-film dispersion combined with probe sonication was used to prepare (ACGs + Rh2)-Lipo. The detailed operations were as follows: 45 mg SPC, 12 mg Rh2, and 2 mg ACGs were co-dissolved in 5 mL ethanol, followed by evaporation at 45 °C for 15 min to form a dry film. Then, the film was hydrated with 3 mL deionized water, and the mixture was performed to a probe sonication for 15 min at 475 W (1 s on, 1 s off, Ningbo Xinzhi Biotechnology Co., Ltd., Ningbo, PR China) to obtain (ACGs + Rh2)-Lipo. The preparation of Rh2-Lipo was the same as (ACGs + Rh2)-Lipo, only without ACGs.

Fluorescent probe DiR labeled liposomes were prepared in accordance with the preparation method of corresponding liposome described above, except appropriate DiR was added to organic solvents.

### 2.5. Characterization of liposomes

Based on dynamic light scattering (DLS), the mean size, polydispersity index (PDI), and zeta potential of liposomes was measured by the Zetasizer Nano ZS instrument (Malvern Instruments, Malvern, UK) at 25 °C in triplicate and with 12 runs.

The morphological characteristics of liposomes were evaluated by a JEM-1400 transmission electron microscope (TEM; JEOL, Tokyo, Japan) after seven times dilution with deionized water and stained with 2% uranium acetate.

The drug-loading content (DLC) and encapsulation efficiency (EE) were calculated based on the total mass of liposomes, the total mass of ACGs and free ACGs in liposomes. The weight of lyophilized powder of liposomes was the total mass of liposomes. The supernatant was taken for high-performance liquid chromatography (HPLC) analysis to obtain the total ACGs in ACGs-Lipo and (ACGs + Rh2)-Lipo after diluted 10 times with chromatographic methanol and centrifuged at 13,000 rpm for 10 minutes. 0.4 mL ACGs-Lipo and (ACGs + Rh2)-Lipo were placed in the Ultracentrifugal filter (NMWL 10 k, Millipore, Billerica, MA), then centrifuged at 13,000 rpm for 30 minutes, respectively. The filtrate was used for HPLC analysis to obtain free ACGs in the liposomes. DLC and EE were calculated according to the following equations:

(1) $$\text{DLC}\left(\text{\%}\right)\;=\;\frac{W_{t}-W_{f}}{W}\times \text{100\%}$$

(2) $$\text{EE}\left(\text{\%}\right)\text{=}\frac{W_{t}-W_{f}}{W_{t}}\text{×100\%}$$

where *W* is the weight of the lyophilized powder, and *W_t_* and *W_f_* are the weight of total ACGs and free ACGs in liposomes, respectively.

### 2.6. HPLC analysis of Rh2 and ACGs

High-performance liquid chromatography (HPLC, DIONEX Ultimate 3000, Sunnyvale, CA) was used to accurately determine the content of Rh2 and ACGs in liposomes with the same chromatographic column (Venusil XBP C18 (L), 4.6 mm × 250 mm, 5 μm) at 25 °C. As the most main component in ACGs, squamocin was used as a quantitative indicator for the determination of ACGs with the detection wavelength being 208 nm. The mobile phase consisted of 0.3% phosphoric acid solution and acetonitrile (3:7, v/v) with 1.0 mL/min of the flow rate (Ao et al., 2021). The mobile phase was composed of acetonitrile and water (v/v 63/37) with 1.0 mL/min of the flow rate and 203 nm of the detection wavelength for the determination of Rh2.

### 2.7. The stability of liposomes

The liposome solution was mixed with 1.8% NaCl solution and 10% glucose solution at a volume ratio of 1:1, and mixed with PBS, artificial gastric juice, artificial intestinal juice and plasma at a volume ratio of 1:4. The particle size of the obtain six mixed solutions was measured as the initial particle size for 0 h. Then, the six mixed solutions were incubated at 37 °C, and observed for turbidity, precipitation and other visible changes at the set time point, and the particle size was measured by sampling. The above experiment was repeated three times.

### 2.8. *In vitro* release behavior

To simulate the release behavior of ACGs *in vivo*, PBS (pH = 7.4, 0.1 mol/L) was used as a release medium. Three aliquots of ACGs-Lipo and (ACGs + Rh2)-Lipo (3.0 mL, 0.8 mg/mL, calculated by ACGs) was placed into dialysis bags with a molecular weight cut-off of 8000–10,000 (Spectrum Labs, Rancho Dominguez, CA) and dialyzed against 1 L PBS at 37 °C with stirring at 200 rpm. At specific timepoints, 50 μL dialysate was sampled and replaced with an equal volume of fresh PBS. The release medium was updated every day. ACGs content in the dialysate was determined by HPLC after centrifugation at 13,000 rpm for 10 min.

### 2.9. The evaluation of *in vitro* cytotoxicity

The *in vitro* cytotoxicity of the liposomes and the synergistic inhibition of ACGs and Rh2 were determined by MTS kit (Promega, Madison, WI). 0.1 mL U87 MG cells suspension was dispersed in 96-well plate (5 × 10^6^ cells/well) and incubated at 37 °C. After 24 hours, the supernatant was replaced by 0.15 mL different concentrations of free ACGs and free Rh2 solution with 0.5% DMSO as the negative control or replaced by different concentrations of Rh2-Lipo, ACGs-Lipo, and (ACGs + Rh2)-Lipo with blank MEM medium as the negative control. The other three holes without seeding cells were left as blank control, and other operations were the same as the corresponding negative control. After incubating the drug with cells for 72 hours, 20 μL MTS was added and incubated for three hours. The absorbance was determined at 490 nm through a plate reader (Biotek Synergy H1, Winooski, VT). The cell viability rate was calculated as follows. The half inhibitory concentration (IC_50_) value was calculated using GraphPad Prism Software, Version 5 (GraphPad Software, Inc., La Jolla, CA).

(3) $$\text{Cell viability rate }(\%)=\frac{{\text{OD}}_{e}-{\text{OD}}_{b}}{{\text{OD}}_{n}-{\text{OD}}_{b}}\times 100\%$$

where OD_e_, OD_b_, and OD_n_ are the mean optical densities of the experimental group, blank control group, and negative control group, respectively.

### 2.10. *In vivo* antitumor efficacy and biodistribution in U87 MG tumor‑bearing mice

BALB/c nude mice are immunodeficient mice that are suitable for establishing tumor-bearing mouse models of human derived tumor cells, and were therefore used as the animal model for this study. The logarithmic U87 MG cells (5 × 10^6^cells/mouse) were inoculated into the right armpit of five female nude mice. The tumors (about 1000 mm^3^) were dissected, washed off the blood and tissue on the surface with normal saline, and then cut into 2 × 2 × 2 mm tumor small pieces. The tumor small pieces were transplanted subcutaneously to the right armpit of 30 female nude mice to establish U87 MG tumor‑bearing mice model. After the tumor volume was about 100 mm^3^, the mice were randomly divided into five groups as follows: negative control group (normal saline), positive control group (TMZ, 25 mg/kg), Rh2-Lipo (1.2 mg/kg, calculated by Rh2), ACGs-Lipo (0.2 mg/kg, calculated by ACGs), and (ACGs + Rh2)-Lipo (0.2 + 1.2 mg/kg, calculated by ACGs and Rh2, respectively). The positive control group was given orally TMZ aqueous solution (25 mg/kg) every day for 15 times, and the other groups were given intravenously every other day for eight times. During the administration, the tumor volume and body weight of mice were monitored every two days. Twenty-four hours after the last administration, the mice were euthanized by 100% CO_2_, then the tumor, liver, and spleen were taken out and weighed. The tumor inhibition rate (TIR), liver index, and spleen index were calculated according to the following formula:

(4) $$\text{TIR}(\%)=\frac{W_{\text{nt}}-W_{\text{et}}}{W_{\text{nt}}}\times 100\%$$

(5) $$\text{Liver index }(\%)=\frac{W_{\text{el}}}{W_{\text{nl}}}\times 100\%$$

(6) $$\text{Spleen index }(\%)=\frac{W_{\text{es}}}{W_{\text{ns}}}\times 100\%$$

where *W*_et_, *W*_el_, and *W*_es_ are the average tumor, liver, and spleen weights of the experimental group, respectively, and *W*_nt_, *W*_nl_, and *W*_ns_ are the average tumor, liver, and spleen weights of the negative control group, respectively.

At the last administration, ACGs-Lipo and (ACGs + Rh2)-Lipo were replaced by DiR labeled DiR-ACGs-Lipo and DiR-(ACGs + Rh2)-Lipo, respectively. The *ex vivo* distribution of liposomes in tumor bearing mice was observed by IVIS Living Image@4.4 (Caliper Life Sciences, Hopkinton, MA). Twenty-four hours after administration, the mice were euthanized by 100% CO_2_, and the tumor and main organs of the mice were dissected and imaged as described above to observe the *ex vivo* distribution of liposomes. The fluorescence intensity in tumors and main organs was quantitatively analyzed by Living Image software (version 4.2). The relative tumor targeting index (RTTI) was calculated by following formula:

(7) $$\text{RTTI}=\frac{{\text{ROI}}_{t}}{{\text{ROI}}_{l}}$$

where ROI_t_ and ROI_l_ are the mean fluorescence intensity of the tumor and liver, respectively.

In the above *in vivo* animal research, the only trauma to the experimental mice was subcutaneous injection and tail vein injection, which was less painful, so no anesthesia was administered. In addition, in order to minimize the suffering of mice as much as possible, a clean and comfortable living environment was provided, ensuring sufficient food and water sources.

### 2.11. Statistical analysis

All data are presented as the mean ± SD and were analyzed by IBM SPSS Statistics software, Version19 (IBM Corporation, Armonk, NY). Means were considered significantly different when *p* < .05.

## 3. Results and discussion

### 3.1. Preparation of ACGs-Lipo

The common preparation methods for liposomes include thin-film dispersion method, ultrasonic-dripping method, stirring-dropping method, and reverse-phase evaporation method (Alavi et al., 2022; Huang & Chen, 2023; Munot et al., 2023). The reverse-phase evaporation method is more suitable for loading water-soluble drugs, so the other three methods were selected to prepare ACGs-Lipo and the optimal preparation method for ACGs-Lipo was selected based on the average particle size and PDI of the liposomes. As shown in Table 1, the preparation method has a great influence on the characteristics of ACGs-Lipo. The mean particle size of ACGs-Lipo by thin-film dispersion method (98.5 ± 2.1 nm) and stirring-dropping method (97.7 ± 1.3 nm) was the smallest while the latter has a smaller PDI value of 0.20 ± 0.02. The effect of the water phase used on ACGs-Lipo was also investigated. When deionized water was used as the water phase, the liposomes exhibited a smaller mean particle size (93.1 ± 0.5 nm) and PDI (0.11 ± 0.03) compared to PBS (pH 7.4) as the water phase. As a result, stirring-dropping method with deionized water as the water phase was selected.

**Table 1.** Optimization of the preparation method of ACGs-Lipo (*n* = 3, mean ± SD).

Preparation method	Aqueous phase	Average particle size (nm)	PDI
Thin-film dispersion	PBS (pH 7.4)	98.5 ± 2.1	0.33 ± 0.00
Ultrasonic-dripping	PBS (pH 7.4)	159.0 ± 0.1	0.17 ± 0.03
Stirring-dropping	PBS (pH 7.4)	97.7 ± 1.3	0.20 ± 0.02
Stirring-dropping	Deionized water (pH 6.0)	93.1 ± 0.5	0.11 ± 0.03

**Figure 1.**
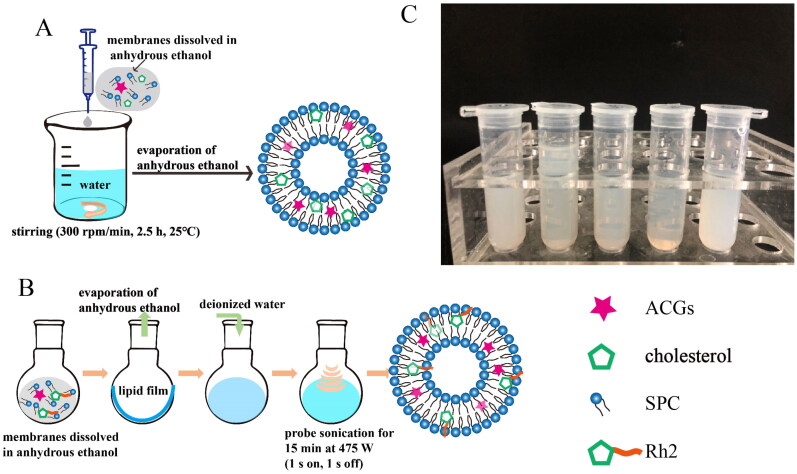
Preparation of liposomes. The optimal preparation process of ACGs-Lipo (A) and (ACGs + Rh2)-Lipo (B). (C) The appearance of (ACGs + Rh2)-Lipo at different concentrations of phospholipid (from left to right: 8 mg/mL, 10 mg/mL, 12 mg/mL, 15 mg/mL, and 18 mg/mL). All details on A and B were created by the author Hui Ao.

In addition, other process conditions affecting the characteristics of ACGs-Lipo were investigated, including stirring time, the ratio of phospholipid to cholesterol, and the ratio of ACGs to phospholipid. These factors have different effects on the formation of ACGs-Lipo, as detailed in Tables S1–S3. As a consequence, the optimal preparation process of ACGs-Lipo was determined, as described in Sections 2.3 and 3.1 (Figure 1(A)).

**Table 3.** Optimization of the concentration of phospholipid in the preparation of (ACGs + Rh2)-Lipo (*n* = 3, mean ± SD).

Concentration of phospholipid (mg/mL)	Average particle size (nm)	PDI	Zeta potential (mV)
8	289.5 ± 5.3	0.24 ± 0.00	−2.9 ± 0.8
10	112.6 ± 0.0	0.13 ± 0.04	−1.7 ± 0.8
12	115.4 ± 1.3	0.19 ± 0.01	−2.7 ± 0.5
15	79.6 ± 0.7	0.17 ± 0.00	−1.1 ± 0.1
18	199.0 ± 5.1	0.68 ± 0.11	−0.5 ± 0.2

### 3.2. Preparation of (ACGs + Rh2)-Lipo

Similarly, the optimum preparation process for (ACGs + Rh2)-Lipo was explored. The effects of four preparation methods on DLS characterization of (ACGs + Rh2)-Lipo are presented in Table 2. The thin-film dispersion method demonstrated outstanding advantages, with the average particle size of (ACGs + Rh2)-Lipo prepared (approximately 120 nm) being half that of the dropping method (>200 nm). In particular, the thin-film dispersion combined with probe sonication method was able to obtain (ACGs + Rh2)-Lipo with a smaller particle size (112.6 ± 0 nm) and a smaller PDI (0.13 ± 0.04).

**Table 2.** Optimization of the preparation method of (ACGs + Rh2)-Lipo (*n* = 3, mean ± SD).

Preparation method	Average particle size (nm)	PDI	Zeta potential (mV)
Thin-film dispersion combined with probe sonication	112.6 ± 0	0.13 ± 0.04	−1.7 ± 0.8
Thin-film dispersion combined with water bath sonication	128.4 ± 8.3	0.50 ± 0.08	−4.0 ± 0.5
Ultrasonic-dripping	318.0 ± 7.5	0.18 ± 0.02	−2.5 ± 0.6
Stirring-dropping	205.3 ± 3.5	0.23 ± 0.02	−4.2 ± 0.3

Then, the concentration of phospholipid was optimized, and the appearance of the liposome solutions under different phospholipid concentrations is displayed in Figure 1(C). It can be seen that the liposome solutions obtained were more translucent and presented a light blue opalescence at phospholipid concentrations of 10 mg/mL, 12 mg/mL, and 15 mg/mL, especially at 15 mg/mL, where the solution was the most translucent and had the strongest opalescence. The DLS data for the liposomes in Table 3 lead to the same conclusion, with the smallest mean particle size (79.6 ± 0.6 nm) and PDI (0.17 ± 0.00) for (ACGs + Rh2)-Lipo at a phospholipid concentration of 15 mg/mL. Consequently, the best preparation method for (ACGs + Rh2)-Lipo was the thin-film dispersion combined with probe sonication method with a phospholipid concentration of 15 mg/mL (Figure 1(B)).

### 3.3. Characterization of liposomes

**Figure 2.**
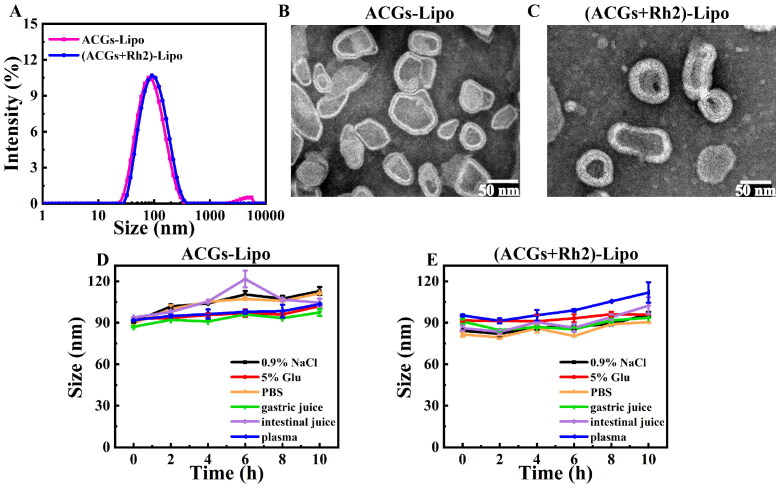
Characterization of liposomes. (A) Particle size distributions of ACGs-Lipo and (ACGs + Rh2)-Lipo. The morphology of ACGs-Lipo (B), and (ACGs + Rh2)-Lipo (C). Particle size change curves of ACGs-Lipo (D) and (ACGs + Rh2)-Lipo (E) in physiological media (*n* = 3, mean ± SD).

The DLS method was used to determine the mean particle size and zeta potential of the liposomes. The particle size distributions of ACGs-Lipo and (ACGs + Rh2)-Lipo are given in Figure 2(A). Under the optimal preparation conditions, the average particle size of ACGs-Lipo was 78.6 ± 1.6 nm with a PDI value of 0.23 ± 0.00 and a zeta potential of −7.5 ± 0.6 mV. The average particle size of (ACGs + Rh2)-Lipo was 86.6 ± 1.2 nm with a PDI value of 0.17 ± 0.04 and a zeta potential of −2.5 ± 0.4 mV. (ACGs + Rh2)-Lipo had a similar mean particle size of 86.6 ± 1.2 nm and zeta potential of 2.5 ± 0.4 mV, with a PDI value of 0.17 ± 0.04, suggesting that both may have similar tumor penetration abilities.

The morphology of ACGs-Lipo and (ACGs + Rh2)-Lipo was observed by TEM, as shown in Figure 2(B,C). Both of them had the typical lipid bilayer structure of liposomes, showing an irregular sphere-like shape, probably due to the larger chemical structure of ACGs as long-chain fatty acids containing lactone rings and tetrahydrofuran rings, which had a slight effect on the morphology of the liposomes after drug loading.

This study was aimed at the anti-tumor study of ACGs, so the DLC and EE mentioned here were calculated based on ACGs. The EE and DLC of ACGs-Lipo were 97.3 ± 0.9% and 4.1 ± 0.7%, respectively, with the DLC of 5.4%. The EE and DLC of (ACGs + Rh2)-Lipo were 97.9 ± 1.2% and 3.2 ± 0.4%, respectively, with the DLC 3.4%. Both liposomes had the similar EE and the similar DLC to the theoretical DLC, indicating that high quality liposomes can be prepared using either cholesterol or Rh2 as the membrane material, further justifying the rationality of Rh2 instead of cholesterol.

### 3.4. The stability of liposomes

In order to select the appropriate administration method and isotonic regulator for ACGs-Lipo and (ACGs + Rh2)-Lipo, liposome solutions were incubated with normal saline, 5% glucose solution, PBS, artificial gastric fluid, artificial intestinal fluid, and plasma at a temperature similar to that of the human body (37 °C). The stability of liposomes in physiological media can be judged according to the change of average particle size of liposomes and the curve of particle size change over time are displayed in Figure 2(D,E). For ACGs-Lipo, except for 6 h incubation with artificial intestinal fluid, the particle size was relatively large but returned to about 100 nm at a later stage. During incubation with other physiological media, the particle size change curve was more gentle, with a particle size increase of about 20 nm. For (ACGs + Rh2)-Lipo, the increase in particle size was only about 10 nm in the other media, except in plasma, where the particle size increased by 17 nm after 10 h of incubation. The results indicated that ACGs-Lipo and (ACGs + Rh2)-Lipo can stably exist in the above six physiological media without aggregation and precipitation, and meet the requirements for oral and intravenous administration.

### 3.5. *In vitro* release behavior

**Figure 3.**
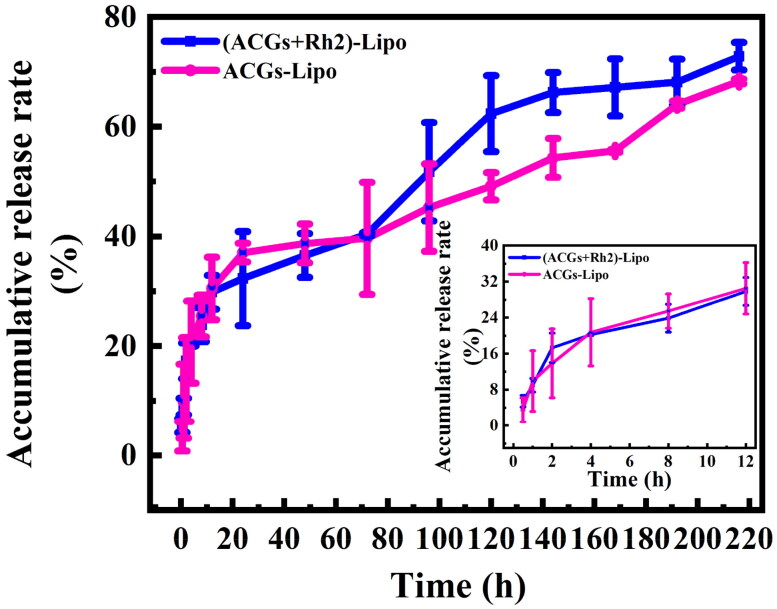
The *in vitro* release profiles of ACGs-Lipo and (ACGs + Rh2)-Lipo (*n* = 3, mean ± SD).

The *in vitro* release of (ACGs + Rh2)-Lipo and ACGs-Lipo in PBS (pH = 7.4) was investigated by dialysis, and the results are exhibited in Figure 3. Both (ACGs + Rh2)-Lipo and ACGs-Lipo showed two-stage release characteristics. The ACGs in the liposomes first displayed a rapid release, due to the portion adsorbed in the outermost layer of the liposomes easily detaching from the liposomes under agitated conditions, followed by a sustained release for the part of the drug loaded into the lipid bilayer. The proportion of burst release of (ACGs + Rh2) Lipo and ACGs-Lipo was consistent, with 20.3% and 20.7% released at four hours, respectively.

From 0 to 72 hours, the release rates of (ACGs + Rh2)-Lipo and ACGs-Lipo were similar, with cumulative release rates of 40.4% and 39.7%, respectively. After 72 hours, the release rate of (ACGs + Rh2)-Lipo was faster than that of ACGs-Lipo, with cumulative release rates of 72.8% and 68.3%, respectively, at 216 h. This difference may be due to the presence of the glucose in the Rh2 structure, which allowed for enhanced interaction of (ACGs + Rh2)-Lipo with the aqueous phase and facilitated the release of ACGs.

### 3.6. The evaluation of *in vitro* cytotoxicity

**Figure 4.**
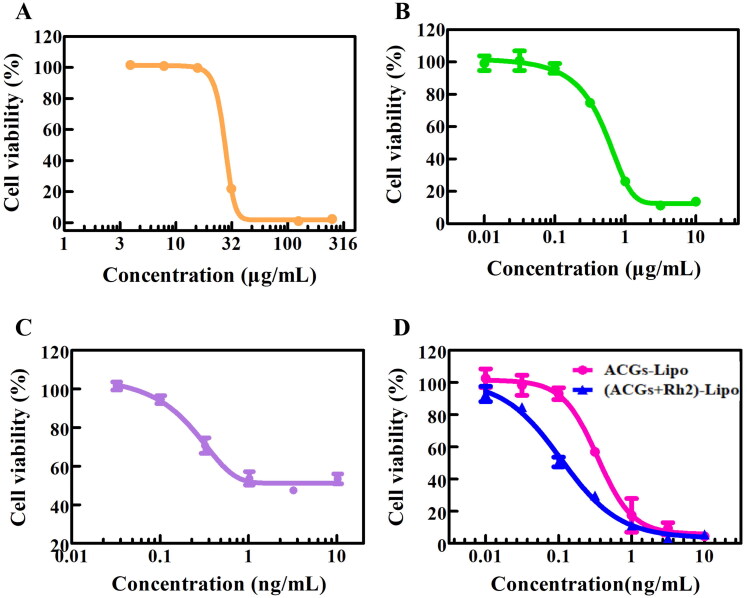
The *in vitro* cytotoxicity of free Rh2 (A), Rh2-Lipo (B), free ACGs (C), and ACGs-Lipo and (ACGs + Rh2)-Lipo (D) against U87 MG cells (*n* = 3, mean ± SD).

The ACGs-Lipo obtained in this study after loading ACGs into liposomes exhibited enhanced cytotoxicity with an IC_50_ (0.34 ± 0.06 ng/mL) that was only one-third of that of free ACGs (1.10 ± 0.80 ng/mL, *p* < .001, Table 4). In addition, ACGs-Lipo showed a better dose-dependent survival curve for U87 MG cells compared to free ACGs (Figure 4(C,D)). This may be due to the higher content of phospholipids in ACGs-Lipo, which are components of cell membranes and can enter cells by membrane fusion. In addition, ACGs-Lipo may also enter the cell mediated by niche and lattice proteins due to its small average particle size (around 80 nm) (Tian et al., 2014; Zhang et al., 2021), which was not affected by the concentration of ACGs as long as sufficient mediator proteins are present. Similarly, free Rh2 (Figure 4(A)) and Rh2-Lipo (Figure 4(B)) had similar phenomena, with IC_50_ for U87 MG cells of 26.31 ± 1.76 μg/mL and 0.47 ± 0.05 μg/mL, respectively (*p* < .001).

**Table 4.** The IC_50_ of free drug and liposomes against U87 MG cells (*n* = 3, mean ± SD).

Group	IC_50_ (μg/mL)
Free Rh2	26.31 ± 1.76
Rh2-Lipo	0.47 ± 0.05***
Free ACGs	0.0011 ± 0.0008###
ACGs-Lipo	0.00034 ± 0.00006
(ACGs + Rh2)-Lipo	0.00011 ± 0.00003&&

*** *p* < .001 vs. free Rh2.

^###^ *p* < .001 vs. free ACGs.

^&&^ *p* < .01 vs. ACGs-Lipo.

The cytotoxicity of the co-loaded liposomes (ACGs + Rh2)-Lipo obtained by replacing cholesterol with Rh2 was compared with that of single-loaded liposomes ACGs-Lipo *in vitro*. As shown in Figure 4(D), the survival rate of U87 MG cells gradually decreased with the increase of the administration concentration of (ACGs + Rh2)-Lipo and ACGs-Lipo, indicating a good dose-dependent relationship. It was evident that the survival curve of (ACGs + Rh2)-Lipo was all below the survival curve of ACGs-Lipo, indicating that (ACGs + Rh2)-Lipo had stronger toxicity *in vitro* at the same concentration of ACGs, with an IC_50_ value of 0.11 ± 0.03 ng/mL (Table 4), which was only one-third of the IC_50_ value of ACGs-Lipo (*p* < .01). Rh2-Lipo has some cytotoxicity of its own, however, Rh2-Lipo had a 95% cell survival rate and negligible effect on the cytotoxicity of (ACGs + Rh2)-Lipo due to the ng/mL level of administration of ACGs (Figure 4(B)). The strong synergistic inhibitory effect of ACGs and Rh2 on U87 MG cells was elucidated.

### 3.7. *In vivo* antitumor efficacy and biodistribution in U87 MG tumor‑bearing mice

**Figure 5.**
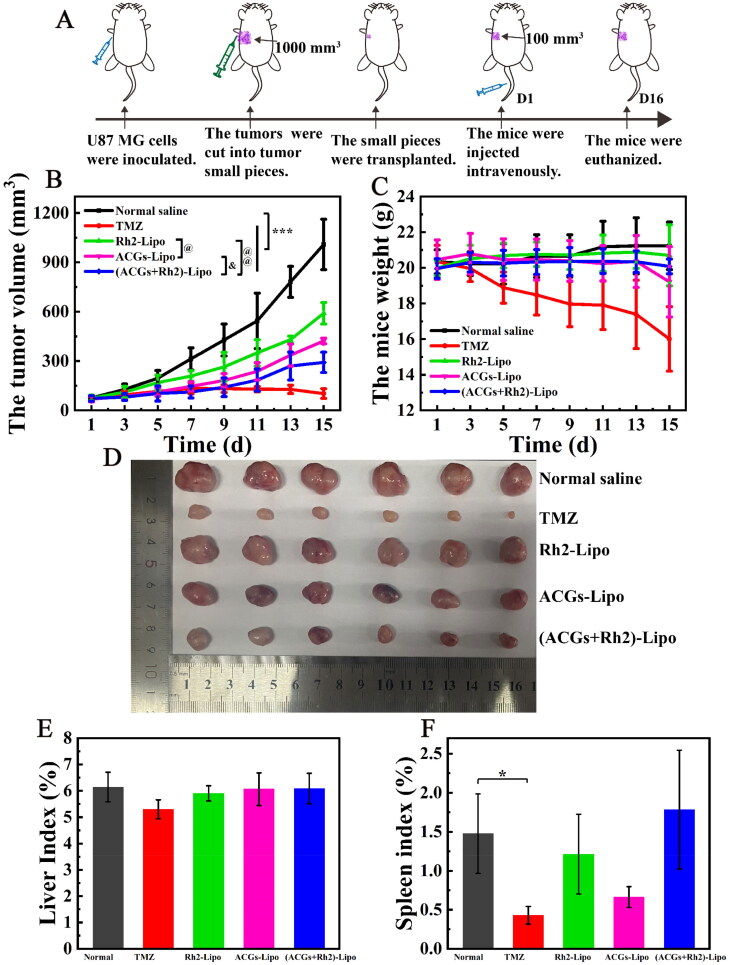
*In vivo* antitumor efficacy in U87 MG tumor‑bearing mice. (A) Schematic diagram of the *in vivo* antitumor study procedure. (B) The change curve of the mouse tumor volume. (C) The change curve of the mouse body weight. (D) The optical photos of tumors at the end of the experiment. (E) The liver index of the mouse in each group. (F) The spleen index of the mouse in each group. Data represent the mean ± SD (*n* = 6).

The synergistic effect of ACGs and Rh2 on anti-glioma *in vivo* was investigated using U87 MG tumor-bearing mice as a model, and the experimental procedure is shown in Figure 5(A). The tumor growth curve of mice in each group during administration is presented in Figure 5(B). The slope of the tumor growth curve in the negative group increased gradually, and the tumor volume on the 15th day after administration was 12 times the initial volume, indicating that the tumor growth rate of the tumor-bearing mice continued to accelerate without any intervention. The tumor volume in the other administration groups was significantly suppressed (*p* < .001), with the positive group having the smallest tumor volume, reflecting the place of TMZ in the treatment of glioma. The tumor of tumor‑bearing mice in the co-loaded liposomes (ACGs + Rh2)-Lipo group grew the slowest among the experimental groups, and the tumor volume on day 15 was both significantly smaller than those of single-loaded liposomes Rh2-Lipo and ACGs-Lipo (*p* < .05), suggesting that ACGs and Rh2 have good synergistic anti-tumor effects. At the termination of the experiment, the tumors of mice in each group were dissected and weighed, and the tumor suppression rate was calculated (Table 5). The relative size of tumors in each group of mice was consistent with the pattern of tumor volume described above. The TIR of the co-loaded group (ACGs + Rh2)-Lipo was 72.9 ± 5.4%, which was significantly higher than that of the Rh2-Lipo (40.3 ± 8.4%, *p* < .01) and ACGs-Lipo (60.5 ± 5.4%, *p* < .05). The optical photographs of tumors in each group at the end of the experiment are given in Figure 5(D), from which the synergistic effect of (ACGs + Rh2)-Lipo may be visualized.

**Table 5.** The anti-glioma activity and changes in body weight of mice in each group (*n* = 6, mean ± SD).

Group	Tumor weight (g)	Tumor inhibition rate (%)	Mice body weight at the beginning of the experiment (g)	Mice body weight after dissection of the tumor (g)	Reduced body weight (g)
Normal saline	1.32 ± 0.16		20.3 ± 0.7	19.9 ± 1.3	0.4
TMZ	0.13 ± 0.06***	90.4 ± 4.6	20.4 ± 0.9	15.9 ± 1.9	4.5
Rh2-Lipo	0.79 ± 0.11^***,^^###^	40.3 ± 8.4###	20.0 ± 0.4	19.9 ± 1.7	0.1
ACGs-Lipo	0.52 ± 0.07^***,^##^,^@	60.5 ± 5.4###^,^@	20.5 ± 1.1	18.7 ± 2.0	1.8
(ACGs + Rh2)-Lipo	0.36 ± 0.07^***,^#^,^@@^,^&	72.9 ± 5.4##^,^@@^,^&	20.0 ± 0.6	19.7 ± 0.4	0.3

*** *p* < .001 vs. normal saline.

^###^ *p* < .001.

^##^ *p* < .01.

^#^ *p* < .05 vs. TMZ.

^@@^ *p* < .01.

^@^ *p* < .05 vs. Rh2-Lipo.

^&^ *p* < .05 vs. ACGs-Lipo.

It is noteworthy that the addition of Rh2 did effectively improve the TIR of ACGs in this study, but this increase was still unsatisfactory. The TIR of (ACGs + Rh2)-Lipo was 72.9 ± 5.4%, less than 80%. We speculate that this limited increase was due to the fact that the model of the efficacy study was U87 MG tumor-bearing nude mice, which were immunodeficient, resulting in a limitation of Rh2 to enhance the anti-tumor activity of ACGs by modulating the immune function of the body.

In addition, the weight change of the mice was also monitored during the experiment, and the change curves are illustrated in Figure 5(C). The decrease in body weight of the mice in the positive group was the most obvious, with a body weight of only 16.0 g on day 15. The body weight of mice in ACGs-Lipo group decreased significantly at the late stage of the experiment, indicating the side effect of ACGs themselves. While the body weight of Rh2-Lipo group and (ACGs + Rh2)-Lipo group increased slightly, suggesting that this adverse effect on body weight could be mitigated by co-administration of Rh2 and ACGs. To more intuitively compare the changes in body weight of the mice before and after administration, the weight of tumor itself was excluded for comparison, and the specific data are presented in Table 5. The body weight loss of mice in the positive group was the most obvious, with a decrease of 4.5 g compared to the initial body weight, equivalent to a quarter of the mice’s own body weight, implying a greater toxic effect of TMZ. Among the experimental groups, only the ACGs-Lipo group had the most significant weight loss of 1.8 g. The Rh2-Lipo and (ACGs + Rh2)-Lipo groups had no obvious weight loss due to the presence of Rh2, demonstrating that Rh2 can attenuate the side effects of ACGs themselves.

In addition to comparing the safety of each group by the body weight of the mice, the liver index and spleen index of mice were also calculated, as shown in Figure 5(E,F). Except for the positive group, where the spleen index was significantly different from that of the negative group (*p* < .05), the liver index and spleen index of all other groups were not significantly different from that of the negative group (*p* > .05), indicating that TMZ had some damage to the spleen, while the liver and the spleen were well tolerated to ACGs with Rh2. In this study, TMZ had the most significant inhibitory effect on glioma, with a TIR of 90.4 ± 4.6%, but it also had the greatest effect on the body weight and the spleen of mice. The co-loaded drug delivery system constructed by AGGs and Rh2 in this study exhibited good anti-glioma activity and safety, which can provide a new option for the treatment of glioma.

**Figure 6.**
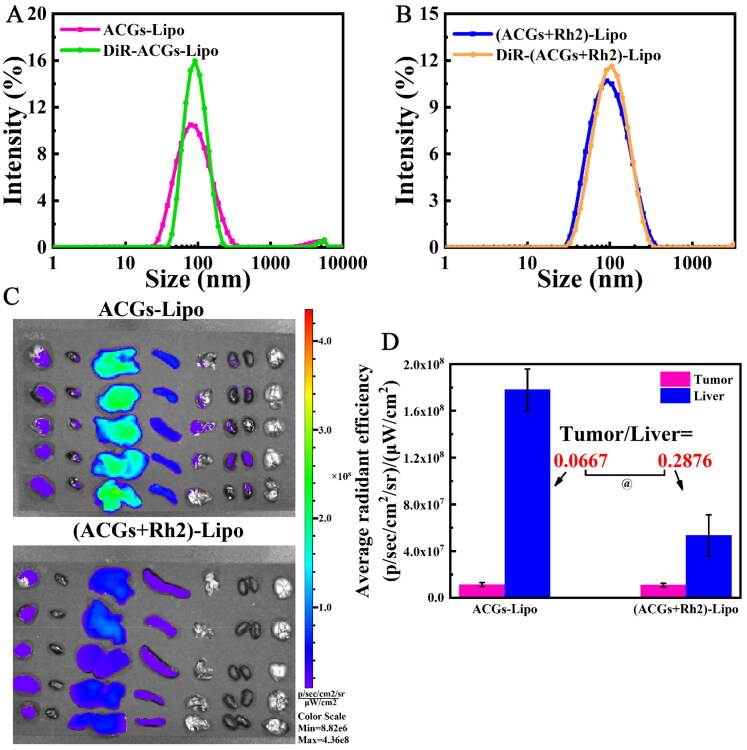
*In vivo* biodistribution in U87 MG tumor‑bearing mice. Comparison of particle size distribution after DiR-labeling of ACGs-Lipo (A) and (ACGs + Rh2)-Lipo (B). (C) The *ex vivo* distribution of DiR-ACGs-Lipo and DiR-(ACGs + Rh2)-Lipo in U87 MG tumor-bearing mice 24 hours after administration (*n* = 5). (D) The fluorescence intensity in tumor and liver of ACGs-Lipo and (ACGs + Rh2)-Lipo and their RTTI (*n* = 5, mean ± SD).

The synergistic effect of ACGs and Rh2 may also result from the targeting of Rh2 in addition to the immunomodulatory effect of Rh2 as described above; therefore, the tumor delivery efficiency of (ACGs + Rh2)-Lipo and ACGs-Lipo was compared. First, the fluorescent probes DiR labeled DiR-ACGs-Lipo and DiR-(ACGs + Rh2)-Lipo were prepared by the same preparation method as ACGs-Lipo and (ACGs + Rh2)-Lipo, respectively, with the aim of comparing the changes in the surface properties of the liposomes before and after loading with DiR. The comparison of the particle size distribution of the four liposomes is displayed in Figure 6(A,B). It can be visualized that the average particle size of both ACGs-Lipo and (ACGs + Rh2)-Lipo increased slightly after loading DiR, but with a narrow particle size distribution and a small average particle size (about 90 nm). It may be that the addition of DiR makes the lipid bilayer slightly larger.

After 24 hours of administration of DiR-labeled liposomes, the *ex vivo* distribution of DiR-ACGs-Lipo and DiR-(ACGs + Rh2)-Lipo is shown in Figure 6(C), both of which were mainly distributed in liver, followed by spleen, with a small amount of distribution in tumor. Semi-quantitative analysis of tumor and liver fluorescence was performed, and the data are exhibited in Figure 6(D). Due to differences in the preparation process, the intravenous doses of DiR were inconsistent between the two. Therefore, the ratio of tumor to liver fluorescence values (RTTI) was compared, which was 0.0667 for the DiR-ACGs-Lipo group and 0.2876 for DiR-(ACGs + Rh2)-Lipo. It can be seen that after co-loading of ACGs and Rh2, the tumor delivery efficiency was improved by 3.3 times, which further explains that the TIR of (ACGs + Rh2)-Lipo was significantly higher than that of ACGs-Lipo (*p* < .05). The tumor-targeting properties of Rh2 embodied were reported to be due to the glucose content of the Rh2 structure, which was able to avoid recognition by MPS, prolong blood circulation time, and also specifically recognize and bind with the glucose transporter on the surface of tumor cells, thereby increasing their uptake by tumor cells (Hong et al., 2019, 2020). In summary, the enhanced anti-tumor activity and safety of (ACGs + Rh2)-Lipo was due to the potent anti-tumor activity of ACGs itself and the certain anti-tumor activity, active targeting and immunomodulatory effects of Rh2.

## 4. Conclusions

ACGs have received widespread attention for their unique anti-tumor mechanism and powerful anti-tumor activity, but the current bottlenecks limiting their clinical application are their high toxicity. The current research mainly focuses on the construction of tumor targeted delivery system to reduce its damage to normal tissues, which relies on targeted polymers with high technical difficulty and high developmental components. In addition, there are fewer studies on the combination use of ACGs. In this study, a co-loaded liposome system of Rh2 and ACGs was successfully constructed by replacing cholesterol with Rh2, which has its own biological activity. The system (ACGs + Rh2)-Lipo not only alleviated the toxicity of ACGs to the organism, but also enhanced their anti-tumor activity. (ACGs + Rh2)-Lipo had a small mean particle size (86.6 ± 1.2 nm), high EE (97.9 ± 1.2%) and good stability. *In vitro* and *in vivo* pharmacodynamic studies demonstrated good synergistic inhibition of U87 MG cells by ACGs and Rh2. Compared to the single-loaded liposome ACGs-Lipo, (ACGs + Rh2)-Lipo had three times the tumor targeting capacity (RTTI, 0.2876) and 1.2 times the anti-glioma activity (TIR, 72.9%). The successful construction of (ACGs + Rh2)-Lipo is expected to break through their bottleneck and can also provide new ideas for the delivery of other chemotherapeutic drugs, which is of great scientific significance.

## Supplementary Material

Supplemental Material

## Data Availability

The data supporting this work are accessible upon reasonable request from the corresponding author.

## References

[CIT0001] Aguilar-Hernández G, Vivar-Vera MdLÁ, García-Magaña MdL, et al. (2020). Ultrasound-assisted extraction of total acetogenins from the soursop fruit by response surface methodology. Molecules 25:1. doi: 10.3390/molecules25051139. PMC7179111 32138341

[CIT0002] Alavi SE, Raza A, Koohi Moftakhari Esfahani M, et al. (2022). Carboplatin niosomal nanoplatform for potentiated chemotherapy. J Pharm Sci 111:3029–12. doi: 10.1016/j.xphs.2022.06.002. 35675875

[CIT0003] Ao H, Li HW, Lu LK, et al. (2021). Sensitive tumor cell line for annonaceous acetogenins and high therapeutic efficacy at a low dose for choriocarcinoma therapy. J Biomed Nanotechnol 17:2062–70. doi: 10.1166/jbn.2021.3175. 34706806

[CIT0004] Chen C, Lv Q, Li Y, et al. (2021). The anti-tumor effect and underlying apoptotic mechanism of ginsenoside Rk1 and Rg5 in human liver cancer cells. Molecules 26:3926. doi: 10.3390/molecules26133926. 34199025 PMC8271777

[CIT0005] Chian S, Zhao Y, Xu M, et al. (2019). Ginsenoside Rd reverses cisplatin resistance in non-small-cell lung cancer A549 cells by downregulating the nuclear factor erythroid 2-related factor 2 pathway. Anticancer Drugs 30:838–45. doi: 10.1097/CAD.0000000000000781. 31415285

[CIT0006] Deepak P, Kumar P, Arya DK, et al. (2023). c(RGDfK) anchored surface manipulated liposome for tumor-targeted tyrosine kinase inhibitor (TKI) delivery to potentiate liver anticancer activity. Int J Pharm 642:123160. doi: 10.1016/j.ijpharm.2023.123160. 37379892

[CIT0007] Han Z, Cheng S, Dai D, et al. (2023). The gut microbiome affects response of treatments in HER2-negative advanced gastric cancer. Clin Transl Med 13:e1312. 37381590 10.1002/ctm2.1312 PMC10307992

[CIT0008] Hong C, Liang J, Xia J, et al. (2020). One stone four birds: a novel liposomal delivery system multi-functionalized with ginsenoside Rh2 for tumor targeting therapy. Nanomicro Lett 12:129. 34138128 10.1007/s40820-020-00472-8 PMC7770862

[CIT0009] Hong C, Wang D, Liang J, et al. (2019). Novel ginsenoside-based multifunctional liposomal delivery system for combination therapy of gastric cancer. Theranostics 9:4437–49. doi: 10.7150/thno.34953. 31285771 PMC6599661

[CIT0010] Hong J, Li Y, Li Y, et al. (2016). Annonaceous acetogenins nanosuspensions stabilized by PCL–PEG block polymer: significantly improved antitumor efficacy. Int J Nanomedicine 11:3239–53. doi: 10.2147/IJN.S108143. 27486323 PMC4957684

[CIT0011] Hong J, Li Y, Xiao Y, et al. (2016). Annonaceous acetogenins (ACGs) nanosuspensions based on a self-assembly stabilizer and the significantly improved anti-tumor efficacy. Colloids Surf B Biointerfaces 145:319–27. doi: 10.1016/j.colsurfb.2016.05.012. 27209384

[CIT0012] Hong J, Sun Z, Li Y, et al. (2017). Folate-modified annonaceous acetogenins nanosuspensions and their improved antitumor efficacy. Int J Nanomedicine 12:5053–67. doi: 10.2147/IJN.S134284. 28765708 PMC5523974

[CIT0013] Huang J, Chen J. (2023). Pharmacokinetics and pharmacodynamic evaluation of hyaluronic acid-modified imatinib-loaded PEGylated liposomes in CD44-positive Gist882 tumor-bearing mice. J Liposome Res 34:1–16. 37401372 10.1080/08982104.2023.2228888

[CIT0014] Huang WC, Huang TH, Yeh KW, et al. (2021). Ginsenoside Rg3 ameliorates allergic airway inflammation and oxidative stress in mice. J Ginseng Res 45:654–64. doi: 10.1016/j.jgr.2021.03.002. 34764720 PMC8569325

[CIT0015] Jin X, Yang Q, Cai N. (2018). Preparation of ginsenoside compound-K mixed micelles with improved retention and antitumor efficacy. Int J Nanomedicine 13:3827–38. doi: 10.2147/IJN.S167529. 30013338 PMC6039058

[CIT0016] Li B, Qi F, Zhu F, et al. (2023). Nanoparticle-based combination therapy enhances fulvestrant efficacy and overcomes tumor resistance in ER-positive breast cancer. Cancer Res 83:2924–37. doi: 10.1158/0008-5472.CAN-22-3559. 37326467

[CIT0017] Li C, Gou X, Gao H. (2021). Doxorubicin nanomedicine based on ginsenoside Rg1 with alleviated cardiotoxicity and enhanced antitumor activity. Nanomedicine 16:2587–604. doi: 10.2217/nnm-2021-0329. 34719938

[CIT0018] Li H, Li Y, Ao H, et al. (2018). Folate-targeting annonaceous acetogenins nanosuspensions: significantly enhanced antitumor efficacy in HeLa tumor-bearing mice. Drug Deliv 25:880–7. doi: 10.1080/10717544.2018.1455761. 29608108 PMC6058653

[CIT0019] Lu H, Zhang Y, Ran S, et al. (2023). Ginsenoside Rg1 alleviates sleep deprivation-induced learning and memory impairment by inhibiting excessive neuronal apoptosis in zebrafish. Neuroreport 34:566–74. doi: 10.1097/WNR.0000000000001926. 37384937

[CIT0020] Lyu X, Xu X, Song A, et al. (2019). Ginsenoside Rh1 inhibits colorectal cancer cell migration and invasion in vitro and tumor growth in vivo. Oncol Lett 18:4160–6. doi: 10.3892/ol.2019.10742. 31579419 PMC6757309

[CIT0021] Manoharan JP, Nirmala Karunakaran K, Vidyalakshmi S, et al. (2023). Computational binding affinity and molecular dynamic characterization of annonaceous acetogenins at nucleotide binding domain (NBD) of multi-drug resistance ATP-binding cassette sub-family B member 1 (ABCB1). J Biomol Struct Dyn 41:821–32. doi: 10.1080/07391102.2021.2013321. 34907862

[CIT0022] Munot NM, Shinde YD, Shah P, et al. (2023). Formulation and evaluation of chitosan–PLGA biocomposite scaffolds incorporated with quercetin liposomes made by QbD approach for improved healing of oral lesions. AAPS PharmSciTech 24:147. doi: 10.1208/s12249-023-02584-x. 37380851

[CIT0023] Nguyen DT, Nguyen TP, Dinh VT, et al. (2023). Potential from synergistic effect of quercetin and paclitaxel co-encapsulated in the targeted folic-gelatin-pluronic P123 nanogels for chemotherapy. Int J Biol Macromol 243:125248. doi: 10.1016/j.ijbiomac.2023.125248. 37307971

[CIT0024] Ohta K, Fushimi T, Okamura M, et al. (2022). Structure–antitumor activity relationship of hybrid acetogenins focusing on connecting groups between heterocycles and the linker moiety. RSC Adv 12:15728–39. doi: 10.1039/d2ra02399g. 35685710 PMC9131733

[CIT0025] Peña-Corona SI, Hernández-Parra H, Bernal-Chávez SA, et al. (2023). Neopeltolide and its synthetic derivatives: a promising new class of anticancer agents. Front Pharmacol 14:1206334. doi: 10.3389/fphar.2023.1206334. 37346293 PMC10280003

[CIT0026] Peng H, Chen L, Deng Y, et al. (2023). Ginsenoside Rh2 mitigates myocardial damage in acute myocardial infarction by regulating pyroptosis of cardiomyocytes. Clin Exp Hypertens 45:2229536. 37395203 10.1080/10641963.2023.2229536

[CIT0027] Schiller J, Zickermann V. (2022). Binding of natural inhibitors to respiratory complex I. Pharmaceuticals 15:1088. doi: 10.3390/ph15091088. 36145309 PMC9503403

[CIT0028] Silva JPN, Pinto B, Monteiro L, et al. (2023). Combination therapy as a promising way to fight oral cancer. Pharmaceutics 15:1653. doi: 10.3390/pharmaceutics15061653. 37376101 PMC10301495

[CIT0029] Tian T, Zhu YL, Zhou YY, et al. (2014). Exosome uptake through clathrin-mediated endocytosis and macropinocytosis and mediating miR-21 delivery. J Biol Chem 289:22258–67. doi: 10.1074/jbc.M114.588046. 24951588 PMC4139237

[CIT0030] Wang W, Guan F, Sagratini G, et al. (2023). Ginsenoside Rd attenuated hyperglycemia via Akt pathway and modulated gut microbiota in streptozotocin-induced diabetic rats. Curr Res Food Sci 6:100491. doi: 10.1016/j.crfs.2023.100491. 37033737 PMC10074500

[CIT0031] Xia J, Ma S, Zhu X, et al. (2022). Versatile ginsenoside Rg3 liposomes inhibit tumor metastasis by capturing circulating tumor cells and destroying metastatic niches. Sci Adv 8:eabj1262. doi: 10.1126/sciadv.abj1262. 35148178 PMC8836824

[CIT0032] Zhang R, Tang L, Zhao B, et al. (2021). A peptide-based small RNA delivery system to suppress tumor growth by remodeling the tumor microenvironment. Mol Pharm 18:1431–43. doi: 10.1021/acs.molpharmaceut.0c01253. 33522823

[CIT0033] Zhu Y, Liang J, Gao C, et al. (2021). Multifunctional ginsenoside Rg3-based liposomes for glioma targeting therapy. J Control Release 330:641–57. doi: 10.1016/j.jconrel.2020.12.036. 33359582

