# Supplementary material for: Enhanced anti-glioma activity of annonaceous acetogenins based on a novel liposomal co-delivery system with ginsenoside Rh2
Source: Drug Deliv. 2024 Mar 31;31(1):2324716. doi: 10.1080/10717544.2024.2324716 (PMC10984232; doi:10.1080/10717544.2024.2324716)
Supplement: Supplemental Material [file IDRD_A_2324716_SM6575.docx]

Supplementary material for

**Enhanced anti-glioma activity of annonaceous acetogenins based a novel liposomal co-delivery system with ginsenoside Rh2**

Hui Ao^1^, Huizhu Song^1^, Jing Li^1^, Xiangtao Wang^2^*

^1^Department of Pharmacy, the Affiliated Wuxi People's Hospital of Nanjing Medical University, Wuxi People's Hospital, Wuxi Medical Center, Nanjing Medical University, No. 299, Qingyang Road, Liangxi District, Wuxi, 214023, PR China.

^2^Institute of Medicinal Plant Development, Chinese Academy of Medical Sciences & Peking Union Medical College, No. 151, Malianwa North Road, Haidian District, Beijing, 100193, PR China

Corresponding author：Xiangtao Wang*

Mailing address: Institute of Medicinal Plant Development, Chinese Academy of Medical Sciences & Peking Union Medical College, No. 151, Malianwa North Road, Haidian District, Beijing, 100193, PR China

Tel.: +86 10 57833266; fax: +86 10 57833266.

E-mail address: xtaowang@163.com (Xiangtao Wang)

**The Supplementary material includes:**

**Figure S1** Original image for Fig 2B

**Figure S2** Original image for Fig 2C

**Table S1** Optimization of stirring time in the preparation of ACGs-Lipo (n=3, mean ± SD)

**Table S2** Optimization of the ratio of phospholipids to cholesterol in the preparation of ACGs-Lipo (n=3, mean ± SD)

**Table S3** Optimization of the ratio of ACGs to phospholipids in the preparation of ACGs-Lipo (n=3, mean ± SD)

**Supplemental Figures**


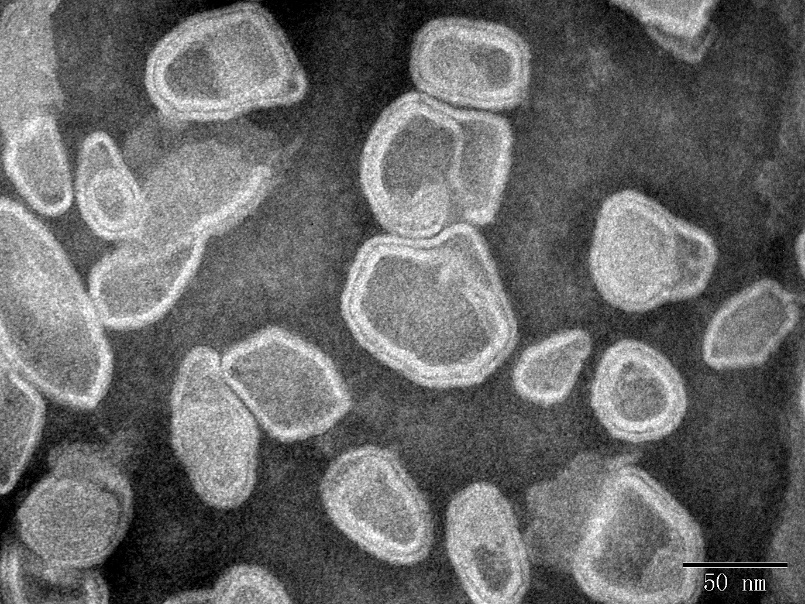


**Figure S1** Original image for Fig 2B


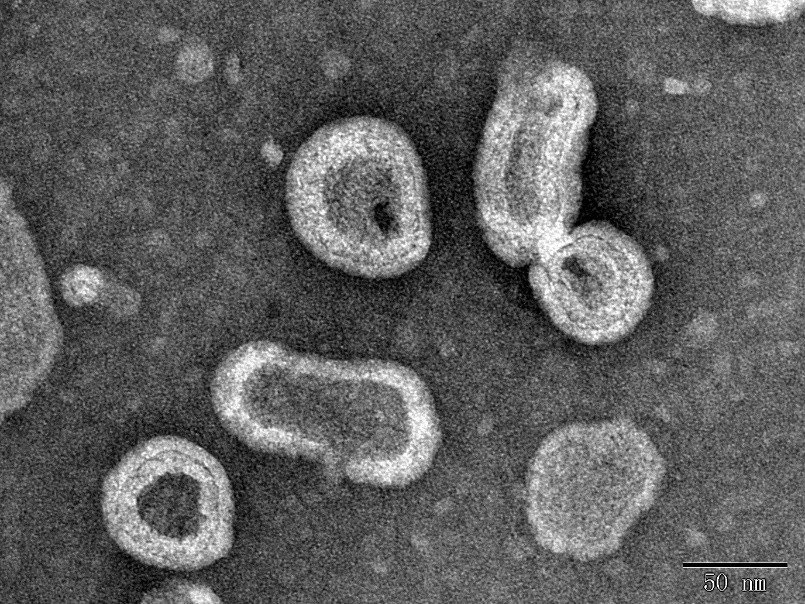


**Figure S2** Original image for Fig 2C

**Supplemental Tables**

**Table S1** Optimization of stirring time in the preparation of ACGs-Lipo (n=3, mean ± SD)

| **Stirring time (h)** | **Average particle size (nm)** | **PDI** | **Zeta potential (mV)** |
| --- | --- | --- | --- |
| 0.5 | 80.9±0.9 | 0.23±0.01 | -5.6±1.0 |
| 1.0 | 85.6±0.3 | 0.22±0.01 | -6.0±0.1 |
| 1.5 | 77.7±0.3 | 0.20±0.01 | -6.9±0.4 |
| 2.0 | 76.0±0.4 | 0.23±0.01 | -8.0±1.6 |
| 2.5 | 60.2±0.2 | 0.24±0.01 | -10.3±1.2 |
| 3.0 | 74.8±0.2 | 0.21±0.01 | -6.3±1.2 |
| 3.5 | 69.6±0.2 | 0.22±0.00 | -9.2±0.6 |
| 4.0 | 63.2±0.1 | 0.23±0.01 | -9.5±0.7 |
| 4.5 | 60.5±0.5 | 0.23±0.00 | -9.1±0.5 |
| 5.0 | 57.6±0.2 | 0.22±0.01 | -10.0±0.7 |

**Table S2** Optimization of the ratio of phospholipids to cholesterol in the preparation of ACGs-Lipo (n=3, mean ± SD)

| **Phospholipids/cholesterol**  **(w/w)** | **Average particle size (nm)** | **PDI** | **Zeta potential (mV)** |
| --- | --- | --- | --- |
| 2:1 | 115.9±1.6 | 0.18±0.02 | -10.1±0.0 |
| 4:1 | 85.8±3.4 | 0.19±0.01 | -6.7±0.5 |
| 6:1 | 65.5±0.7 | 0.22±0.02 | -7.6±1.2 |
| 8:1 | 71.9±0.7 | 0.26±0.00 | -8.2±0.4 |
| 10:1 | 82.9±0.0 | 0.26±0.01 | -11.4±0.6 |

**Table S3** Optimization of the ratio of ACGs to phospholipids in the preparation of ACGs-Lipo (n=3, mean ± SD)

| **ACGs/phospholipids**  **(w/w)** | **Average particle size (nm)** | **PDI** | **Zeta potential (mV)** |
| --- | --- | --- | --- |
| 1:5 | 178.3±3.7 | 0.51±0.02 | -10.0±0.9 |
| 1:10 | 76.3±0.3 | 0.25±0.01 | -10.3±0.7 |
| 1:15 | 64.1±0.6 | 0.24±0.00 | -12.7±3.1 |
| 1:20 | 63.2±1.0 | 0.24±0.01 | -10.0±0.3 |
| 1:25 | 64.7±0.1 | 0.24±0.02 | -10.0±1.0 |
